# Chlorido(pyridine-2-carboximidamide-κ^2^ *N* ^1^,*N* ^2^)zinc(II) chloride dihydrate

**DOI:** 10.1107/S1600536810045848

**Published:** 2010-11-13

**Authors:** Guang-Hua Dong, Rui-De Xue, Jing Li

**Affiliations:** a Jinzhong Vocational & Technical College, Yuci 030600, People’s Republic of China; b School of Chemistry and Chemical Engineering, Shanxi University, Taiyuan 030006, People’s Republic of China

## Abstract

In the title salt, [ZnCl(C_6_H_7_N_3_)_2_]Cl·2H_2_O, the pyridine-2-carboximidamide ligands chelate to the Zn^II^ atom, which is also coordinated by a Cl atom. The Zn^II^ atom shows a trigonal–bipyramidal coordination, with the pyridyl N atoms occupying the axial positions. The cation, anion and water mol­ecules are linked by N—H⋯Cl, N—H⋯O, O—H⋯Cl and O—H⋯O hydrogen bonds into a three-dimensional structure.

## Related literature

For a related compound with a similar coordination mode, see: Li *et al.* (2006 ▶).
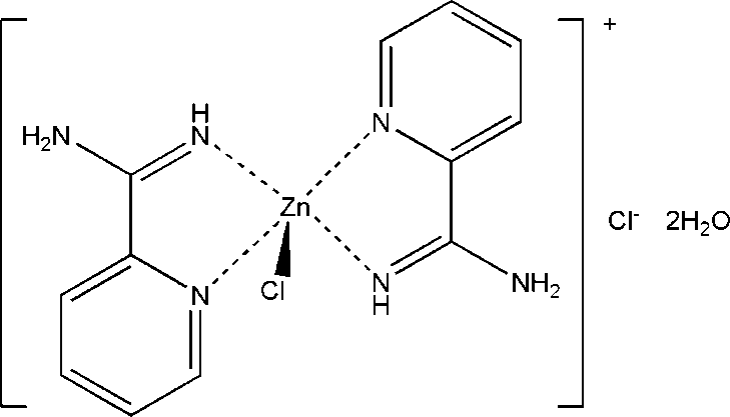


## Experimental

### 

#### Crystal data

[ZnCl(C_6_H_7_N_3_)_2_]Cl·2H_2_O
*M* *_r_* = 414.59
Triclinic, 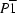

*a* = 7.1658 (14) Å
*b* = 9.7120 (18) Å
*c* = 13.233 (3) Å
α = 92.225 (3)°
β = 96.138 (3)°
γ = 104.302 (3)°
*V* = 885.3 (3) Å^3^
*Z* = 2
Mo *K*α radiation
μ = 1.71 mm^−1^
*T* = 293 K
0.30 × 0.25 × 0.20 mm

#### Data collection

Bruker SMART CCD diffractometer
Absorption correction: multi-scan (*SADABS*; Sheldrick, 1996 ▶) *T*_min_ = 0.629, *T*_max_ = 0.727
3618 measured reflections
3026 independent reflections
2575 reflections with *I* > 2σ(*I*)
*R*_int_ = 0.017

#### Refinement

*R*[*F* ^2^ > 2σ(*F* ^2^)] = 0.042
*wR*(*F* ^2^) = 0.115
*S* = 1.11
3026 reflections
208 parameters
H-atom parameters constrained
Δρ_max_ = 0.44 e Å^−3^
Δρ_min_ = −0.42 e Å^−3^

### 

Data collection: *SMART* (Bruker, 2000 ▶); cell refinement: *SAINT* (Bruker, 2000 ▶); data reduction: *SAINT*; program(s) used to solve structure: *SHELXS97* (Sheldrick, 2008 ▶); program(s) used to refine structure: *SHELXL97* (Sheldrick, 2008 ▶); molecular graphics: *SHELXTL/PC* (Sheldrick, 2008 ▶); software used to prepare material for publication: *SHELXTL/PC*.

## Supplementary Material

Crystal structure: contains datablocks I, global. DOI: 10.1107/S1600536810045848/ng5060sup1.cif

Structure factors: contains datablocks I. DOI: 10.1107/S1600536810045848/ng5060Isup2.hkl

Additional supplementary materials:  crystallographic information; 3D view; checkCIF report

## Figures and Tables

**Table 1:** Hydrogen-bond geometry (Å, °)

*D*—H⋯*A*	*D*—H	H⋯*A*	*D*⋯*A*	*D*—H⋯*A*
N1—H1*A*⋯Cl2	0.86	2.86	3.553 (3)	138
N2—H2*A*⋯O2^i^	0.86	2.13	2.942 (4)	156
N2—H2*B*⋯Cl2^ii^	0.86	2.61	3.434 (3)	160
N4—H4*B*⋯Cl2^iii^	0.86	2.68	3.432 (3)	147
N5—H5*B*⋯Cl2^iii^	0.86	2.51	3.295 (3)	152
N5—H5*C*⋯Cl1^iv^	0.86	2.56	3.289 (3)	144
O1—H1*C*⋯Cl1	0.82	2.64	3.320 (4)	142
O1—H1*D*⋯Cl2	0.82	2.44	3.237 (4)	165
O2—H2*D*⋯Cl2^v^	0.83	2.40	3.182 (4)	157
O2—H2*C*⋯O1	0.83	1.92	2.753 (5)	173

Symmetry codes: (i) 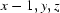; (ii) 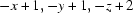; (iii) 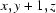; (iv) 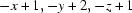; (v) 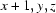.

**Fig. 1.**
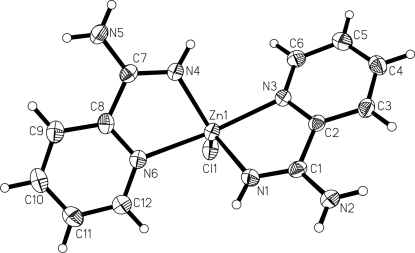
The cation structure, showing the atom-numbering scheme. Displacement ellipsoids were drawn at the 30% probability level.

## supplementary crystallographic information

### Comment

The zinc ion is coordinated *via* two nitrogen of the ligand, two five rings of N1—C1—C2—N3—Zn1 and N4—C7—C8—N6—Zn1 are formed. The torsion angle between the two five-membered rings is 168.41°. The bond lengthes of Zn1—N1 and Zn1—N3 of the compound are 1.980 (3) and 2.201 (3) Å, respectively. The coordination geometry of Zn^2+^ is distorted tetragonal pyramid. This is comparable to the compound 7 reported by Karlin group (Li *et al.*, 2006), which can provide three nitrogen and two oxygen to coordination zinc(II), the average bond length of Zn—N is 2.104 (3) Å. There are four types of hydrogen bond, namely O···H—N, Cl···H—N and O···H—O, Cl···H—O, in the packing structure. These multi hydrogen bond are due to the chloride anions and exogenous water molecules existed in the interspace of compound. By the interactions of hydrogen bond, the infinite three dimensional structure is formed.

### Experimental

Synthesis: Zinc chloride 0.221 g (1.5 mmol) was added to the solution of LiN(SiMe_3_)_2_ (3.0 mmol) and 2-cyanopyridine(0.30 ml, 3.0 mmol) in thf (30 ml) at 195 K. The mixture was stirred for 12 h at ambient temperature, then filtered. The filtrate was concentrated and the residue was crystallized from ethanol at ambient temperature, yielding colorless crystal [ZnCl(C_6_H_7_N_3_)_2_]Cl^.^2H_2_O(0.28 g, 42%). ^1^H NMR([D_6_]DMSO): 7.56(s, 4H, NH), 7.94 (d, J 4.4 Hz, 8H, pyridyl) and 8.73 (d, J 4.4 Hz, 8H, pyridyl). mp 506–507 K.

### Refinement

The water H atoms were found by using fourier difference map and constrained to their related atoms, with O—H distances in the range 0.82 Å and *U*_iso_(H) = 1.5*U*_eq_(O). The other H atoms were placed in geometrically idealized positions and constrained to ride on their parent atoms, with C—H distances in the range 0.93 Å and *U*_iso_(H) = 1.2*U*_eq_(C), N—H distances in the range 0.86 Å and *U*_iso_(H) = 1.2*U*_eq_(N),

### Crystal data

**Table:**

[ZnCl(C_6_H_7_N_3_)_2_]Cl·2H_2_O	*Z* = 2
*M**_r_* = 414.59	*F*(000) = 424
Triclinic, *P*1	*D*_x_ = 1.555 Mg m^−^^3^
*a* = 7.1658 (14) Å	Mo *K*α radiation, λ = 0.71073 Å
*b* = 9.7120 (18) Å	Cell parameters from 1653 reflections
*c* = 13.233 (3) Å	θ = 2.6–25.1°
α = 92.225 (3)°	µ = 1.71 mm^−^^1^
β = 96.138 (3)°	*T* = 293 K
γ = 104.302 (3)°	Block, colorless
*V* = 885.3 (3) Å^3^	0.30 × 0.25 × 0.20 mm

### Data collection

**Table:**

Bruker SMART CCD diffractometer	3026 independent reflections
Radiation source: fine-focus sealed tube	2575 reflections with *I* > 2σ(*I*)
graphite	*R*_int_ = 0.017
ω scans	θ_max_ = 25.0°, θ_min_ = 1.6°
Absorption correction: multi-scan (*SADABS*; Sheldrick, 1996)	*h* = −8→8
*T*_min_ = 0.629, *T*_max_ = 0.727	*k* = −11→11
3618 measured reflections	*l* = −15→13

### Refinement

**Table:**

Refinement on *F*^2^	Primary atom site location: structure-invariant direct methods
Least-squares matrix: full	Secondary atom site location: difference Fourier map
*R*[*F*^2^ > 2σ(*F*^2^)] = 0.042	Hydrogen site location: inferred from neighbouring sites
*wR*(*F*^2^) = 0.115	H-atom parameters constrained
*S* = 1.11	*w* = 1/[σ^2^(*F*_o_^2^) + (0.0652*P*)^2^ + 0.0845*P*] where *P* = (*F*_o_^2^ + 2*F*_c_^2^)/3
3026 reflections	(Δ/σ)_max_ = 0.001
208 parameters	Δρ_max_ = 0.44 e Å^−^^3^
0 restraints	Δρ_min_ = −0.42 e Å^−^^3^

### Special details

**Table:**

Geometry. All e.s.d.'s (except the e.s.d. in the dihedral angle between two l.s. planes) are estimated using the full covariance matrix. The cell e.s.d.'s are taken into account individually in the estimation of e.s.d.'s in distances, angles and torsion angles; correlations between e.s.d.'s in cell parameters are only used when they are defined by crystal symmetry. An approximate (isotropic) treatment of cell e.s.d.'s is used for estimating e.s.d.'s involving l.s. planes.
Refinement. Refinement of *F*^2^ against ALL reflections. The weighted *R*-factor *wR* and goodness of fit *S* are based on *F*^2^, conventional *R*-factors *R* are based on *F*, with *F* set to zero for negative *F*^2^. The threshold expression of *F*^2^ > σ(*F*^2^) is used only for calculating *R*-factors(gt) *etc*. and is not relevant to the choice of reflections for refinement. *R*-factors based on *F*^2^ are statistically about twice as large as those based on *F*, and *R*- factors based on ALL data will be even larger.

### Fractional atomic coordinates and isotropic or equivalent isotropic displacement parameters (Å^2^)

**Table:**

	*x*	*y*	*z*	*U*_iso_*/*U*_eq_	
Zn1	0.56451 (5)	0.85104 (4)	0.73653 (3)	0.04193 (17)	
Cl1	0.84868 (13)	0.83366 (11)	0.67568 (7)	0.0547 (3)	
N1	0.4601 (4)	0.7076 (3)	0.8321 (2)	0.0471 (7)	
H1A	0.3837	0.6272	0.8087	0.056*	
N2	0.4396 (5)	0.6468 (3)	0.9992 (2)	0.0500 (7)	
H2A	0.3596	0.5656	0.9812	0.060*	
H2B	0.4767	0.6715	1.0627	0.060*	
N3	0.6905 (4)	0.9590 (3)	0.8866 (2)	0.0376 (6)	
C1	0.5059 (5)	0.7339 (3)	0.9290 (2)	0.0394 (7)	
C2	0.6376 (4)	0.8761 (3)	0.9628 (2)	0.0354 (7)	
C3	0.7007 (5)	0.9224 (4)	1.0639 (3)	0.0431 (8)	
H3A	0.6638	0.8638	1.1160	0.052*	
C4	0.8217 (5)	1.0602 (4)	1.0854 (3)	0.0490 (9)	
H4A	0.8666	1.0938	1.1526	0.059*	
C5	0.8737 (5)	1.1449 (4)	1.0082 (3)	0.0481 (9)	
H5A	0.9523	1.2369	1.0215	0.058*	
C6	0.8061 (5)	1.0899 (4)	0.9094 (3)	0.0440 (8)	
H6A	0.8428	1.1466	0.8563	0.053*	
N4	0.4526 (4)	1.0103 (3)	0.6875 (2)	0.0439 (7)	
H4B	0.4771	1.0895	0.7238	0.053*	
N5	0.2741 (4)	1.0951 (3)	0.5595 (2)	0.0521 (8)	
H5B	0.3001	1.1789	0.5896	0.063*	
H5C	0.2024	1.0767	0.5018	0.063*	
N6	0.3721 (4)	0.7541 (3)	0.5968 (2)	0.0433 (7)	
C7	0.3450 (5)	0.9937 (4)	0.6023 (2)	0.0395 (7)	
C8	0.2952 (4)	0.8486 (4)	0.5478 (2)	0.0404 (8)	
C9	0.1806 (5)	0.8141 (4)	0.4551 (3)	0.0532 (9)	
H9A	0.1276	0.8811	0.4222	0.064*	
C10	0.1473 (6)	0.6754 (5)	0.4125 (3)	0.0641 (11)	
H10A	0.0700	0.6483	0.3504	0.077*	
C11	0.2283 (6)	0.5785 (5)	0.4621 (3)	0.0643 (11)	
H11A	0.2096	0.4864	0.4337	0.077*	
C12	0.3374 (6)	0.6215 (4)	0.5547 (3)	0.0544 (9)	
H12A	0.3895	0.5557	0.5896	0.065*	
O1	0.8460 (6)	0.5476 (4)	0.8055 (3)	0.1095 (13)	
H1C	0.8918	0.6026	0.7637	0.164*	
H1D	0.7433	0.4933	0.7795	0.164*	
O2	1.1203 (5)	0.4197 (3)	0.8929 (3)	0.0831 (9)	
H2C	1.0437	0.4653	0.8687	0.125*	
H2D	1.1647	0.3878	0.8443	0.125*	
Cl2	0.41075 (19)	0.34820 (11)	0.74501 (8)	0.0690 (3)	

### Atomic displacement parameters (Å^2^)

**Table:**

	*U*^11^	*U*^22^	*U*^33^	*U*^12^	*U*^13^	*U*^23^
Zn1	0.0489 (3)	0.0424 (3)	0.0349 (3)	0.01345 (18)	0.00115 (17)	0.00644 (16)
Cl1	0.0507 (5)	0.0773 (7)	0.0398 (5)	0.0231 (5)	0.0043 (4)	0.0085 (4)
N1	0.0593 (18)	0.0356 (15)	0.0403 (17)	0.0031 (13)	0.0005 (13)	0.0015 (12)
N2	0.068 (2)	0.0389 (16)	0.0420 (17)	0.0115 (14)	0.0069 (14)	0.0090 (13)
N3	0.0405 (14)	0.0374 (15)	0.0357 (15)	0.0117 (12)	0.0031 (11)	0.0043 (11)
C1	0.0448 (18)	0.0355 (18)	0.043 (2)	0.0175 (14)	0.0080 (14)	0.0073 (14)
C2	0.0357 (16)	0.0370 (17)	0.0373 (17)	0.0163 (13)	0.0035 (13)	0.0051 (13)
C3	0.0460 (19)	0.050 (2)	0.0371 (18)	0.0202 (16)	0.0017 (14)	0.0066 (15)
C4	0.049 (2)	0.056 (2)	0.044 (2)	0.0231 (17)	−0.0051 (16)	−0.0092 (17)
C5	0.0380 (18)	0.045 (2)	0.059 (2)	0.0105 (15)	0.0011 (16)	−0.0051 (17)
C6	0.0442 (18)	0.0388 (19)	0.048 (2)	0.0086 (15)	0.0070 (15)	0.0065 (15)
N4	0.0559 (17)	0.0414 (16)	0.0354 (16)	0.0162 (13)	−0.0006 (13)	0.0036 (12)
N5	0.065 (2)	0.0533 (19)	0.0432 (17)	0.0263 (15)	0.0002 (14)	0.0086 (14)
N6	0.0460 (16)	0.0441 (17)	0.0389 (16)	0.0111 (13)	0.0015 (12)	0.0041 (13)
C7	0.0405 (17)	0.0446 (19)	0.0382 (19)	0.0159 (14)	0.0115 (14)	0.0103 (14)
C8	0.0345 (17)	0.054 (2)	0.0325 (17)	0.0084 (15)	0.0075 (13)	0.0063 (15)
C9	0.054 (2)	0.064 (3)	0.042 (2)	0.0182 (18)	0.0002 (16)	0.0044 (18)
C10	0.065 (3)	0.075 (3)	0.045 (2)	0.012 (2)	−0.0090 (18)	−0.008 (2)
C11	0.079 (3)	0.053 (2)	0.056 (3)	0.012 (2)	0.001 (2)	−0.009 (2)
C12	0.064 (2)	0.048 (2)	0.051 (2)	0.0149 (18)	0.0012 (18)	0.0043 (18)
O1	0.119 (3)	0.102 (3)	0.112 (3)	0.045 (2)	−0.011 (2)	0.017 (2)
O2	0.076 (2)	0.075 (2)	0.094 (2)	0.0119 (17)	0.0074 (17)	0.0119 (18)
Cl2	0.1080 (9)	0.0486 (6)	0.0525 (6)	0.0233 (6)	0.0097 (5)	0.0055 (4)

### Geometric parameters (Å, °)

**Table:**

Zn1—N1	1.981 (3)	N4—C7	1.277 (4)
Zn1—N4	2.009 (3)	N4—H4B	0.8600
Zn1—N3	2.201 (3)	N5—C7	1.334 (4)
Zn1—N6	2.210 (3)	N5—H5B	0.8600
Zn1—Cl1	2.3095 (10)	N5—H5C	0.8600
N1—C1	1.288 (4)	N6—C8	1.336 (4)
N1—H1A	0.8600	N6—C12	1.337 (5)
N2—C1	1.328 (4)	C7—C8	1.500 (5)
N2—H2A	0.8600	C8—C9	1.382 (5)
N2—H2B	0.8600	C9—C10	1.394 (6)
N3—C6	1.338 (4)	C9—H9A	0.9300
N3—C2	1.342 (4)	C10—C11	1.375 (6)
C1—C2	1.489 (5)	C10—H10A	0.9300
C2—C3	1.386 (5)	C11—C12	1.371 (5)
C3—C4	1.403 (5)	C11—H11A	0.9300
C3—H3A	0.9300	C12—H12A	0.9300
C4—C5	1.362 (5)	O1—H1C	0.8202
C4—H4A	0.9300	O1—H1D	0.8235
C5—C6	1.382 (5)	O2—H2C	0.8330
C5—H5A	0.9300	O2—H2D	0.8300
C6—H6A	0.9300		
			
N1—Zn1—N4	127.46 (12)	C6—C5—H5A	121.0
N1—Zn1—N3	77.12 (11)	N3—C6—C5	123.1 (3)
N4—Zn1—N3	95.03 (11)	N3—C6—H6A	118.4
N1—Zn1—N6	98.65 (11)	C5—C6—H6A	118.4
N4—Zn1—N6	76.70 (11)	C7—N4—Zn1	119.8 (2)
N3—Zn1—N6	165.87 (10)	C7—N4—H4B	120.1
N1—Zn1—Cl1	116.09 (10)	Zn1—N4—H4B	120.1
N4—Zn1—Cl1	116.45 (9)	C7—N5—H5B	120.0
N3—Zn1—Cl1	98.33 (7)	C7—N5—H5C	120.0
N6—Zn1—Cl1	95.63 (8)	H5B—N5—H5C	120.0
C1—N1—Zn1	120.5 (2)	C8—N6—C12	119.0 (3)
C1—N1—H1A	119.8	C8—N6—Zn1	112.2 (2)
Zn1—N1—H1A	119.8	C12—N6—Zn1	128.6 (3)
C1—N2—H2A	120.0	N4—C7—N5	124.7 (3)
C1—N2—H2B	120.0	N4—C7—C8	116.8 (3)
H2A—N2—H2B	120.0	N5—C7—C8	118.4 (3)
C6—N3—C2	118.9 (3)	N6—C8—C9	122.3 (3)
C6—N3—Zn1	129.3 (2)	N6—C8—C7	114.1 (3)
C2—N3—Zn1	111.8 (2)	C9—C8—C7	123.6 (3)
N1—C1—N2	125.1 (3)	C8—C9—C10	117.6 (4)
N1—C1—C2	116.2 (3)	C8—C9—H9A	121.2
N2—C1—C2	118.6 (3)	C10—C9—H9A	121.2
N3—C2—C3	121.7 (3)	C11—C10—C9	120.1 (4)
N3—C2—C1	114.4 (3)	C11—C10—H10A	119.9
C3—C2—C1	123.9 (3)	C9—C10—H10A	119.9
C2—C3—C4	118.1 (3)	C12—C11—C10	118.2 (4)
C2—C3—H3A	121.0	C12—C11—H11A	120.9
C4—C3—H3A	121.0	C10—C11—H11A	120.9
C5—C4—C3	120.2 (3)	N6—C12—C11	122.7 (4)
C5—C4—H4A	119.9	N6—C12—H12A	118.6
C3—C4—H4A	119.9	C11—C12—H12A	118.6
C4—C5—C6	118.0 (3)	H1C—O1—H1D	109.8
C4—C5—H5A	121.0	H2C—O2—H2D	107.0

### Hydrogen-bond geometry (Å, °)

**Table:**

*D*—H···*A*	*D*—H	H···*A*	*D*···*A*	*D*—H···*A*
N1—H1A···Cl2	0.86	2.86	3.553 (3)	138
N2—H2A···O2^i^	0.86	2.13	2.942 (4)	156
N2—H2B···Cl2^ii^	0.86	2.61	3.434 (3)	160
N4—H4B···Cl2^iii^	0.86	2.68	3.432 (3)	147
N5—H5B···Cl2^iii^	0.86	2.51	3.295 (3)	152
N5—H5C···Cl1^iv^	0.86	2.56	3.289 (3)	144
O1—H1C···Cl1	0.82	2.64	3.320 (4)	142
O1—H1D···Cl2	0.82	2.44	3.237 (4)	165
O2—H2D···Cl2^v^	0.83	2.40	3.182 (4)	157
O2—H2C···O1	0.83	1.92	2.753 (5)	173

Symmetry codes: (i) *x*−1, *y*, *z*; (ii) −*x*+1, −*y*+1, −*z*+2; (iii) *x*, *y*+1, *z*; (iv) −*x*+1, −*y*+2, −*z*+1; (v) *x*+1, *y*, *z*.
